# Yeast Surface Display of Two Proteins Previously Shown to Be Protective Against White Spot Syndrome Virus (WSSV) in Shrimp

**DOI:** 10.1371/journal.pone.0128764

**Published:** 2015-06-17

**Authors:** Vorawit Ananphongmanee, Jiraporn Srisala, Kallaya Sritunyalucksana, Chuenchit Boonchird

**Affiliations:** 1 Department of Biotechnology, Faculty of Science, Mahidol University, Bangkok, Thailand; 2 Shrimp-Virus Interaction Laboratory (ASVI), National Center for Genetic Engineering and Biotechnology (BIOTEC), National Science and Technology Development Agency (NSTDA), Bangkok, Thailand; 3 Center of Excellence for Shrimp Molecular Biology and Biotechnology, Faculty of Science, Mahidol University, Bangkok, Thailand; Uppsala University, SWEDEN

## Abstract

Cell surface display using the yeasts *Saccharomyces cerevisiae* and *Pichia pastoris* has been extensively developed for application in bioindustrial processes. Due to the rigid structure of their cell walls, a number of proteins have been successfully displayed on their cell surfaces. It was previously reported that the viral binding protein Rab7 from the giant tiger shrimp *Penaeus monodon* (*Pm*Rab7) and its binding partner envelope protein VP28 of white spot syndrome virus (WSSV) could independently protect shrimp against WSSV infection. Thus, we aimed to display these two proteins independently on the cell surfaces of 2 yeast clones with the ultimate goal of using a mixture of the two clones as an orally deliverable, antiviral agent to protect shrimp against WSSV infection. *Pm*Rab7 and VP28 were modified by N-terminal tagging to the C-terminal half of **S*. *cerevisiae** α-agglutinin. DNA fragments, harboring fused-gene expression cassettes under control of an alcohol oxidase I *(AOX1)* promoter were constructed and used to transform the yeast cells. Immunofluorescence microscopy with antibodies specific to both proteins demonstrated that mutated *Pm*Rab7 (m*Pm*Rab7) and partial VP28 (pVP28) were localized on the cell surfaces of the respective clones, and fluorescence intensity for each was significantly higher than that of control cells by flow cytometry. Enzyme-linked immunosorbant assay (ELISA) using cells displaying m*Pm*Rab7 or pVP28 revealed that the binding of specific antibodies for each was dose-dependent, and could be saturated. In addition, the binding of m*Pm*Rab7-expressing cells with free VP28, and vice versa was dose dependent. Binding between the two surface-expressed proteins was confirmed by an assay showing agglutination between cells expressing complementary m*Pm*Rab7 and pVP28. In summary, our genetically engineered *P*. *pastoris* can display biologically active m*Pm*Rab7 and pVP28 and is now ready for evaluation of efficacy in protecting shrimp against WSSV by oral administration.

## Introduction

Microbial cell-surface based technology is one approach that allows a target peptide or protein to be presented on the surface of cells for protein engineering and purification. Phage, bacteria, yeasts and other fungi have been used to express target proteins on their surfaces [1]. The first surface display was developed in the mid-1980s by Smith, who displayed peptides and small proteins on the surface of a bacteriophage [2]. Yeast cell surface display is the most useful among various display systems developed so far. It is suitable for many applications including environmental treatments, biocatalysts, food and feed supplements, vaccines, *etc*. The yeasts *S*. *cerevisiae*, *P*. *pastoris* and *Yarrowia lipolytica* have “generally regarded as safe” (GRAS) status, making them acceptable for food and pharmaceutical applications [3]. In a yeast cell surface display system, a target protein is attached to a cell-membrane bound glycosylphosphatidylinositol (GPI)-anchor protein so that it will be presented on the yeast cell wall. For this reason, a number of proteins with sizes varying from 93–136 kDa have been successfully displayed on the yeast cell surface [3].

The yeast cell wall has a mass composition of 30–50% mannoproteins, 30–45% β-1,3 glucans, 5–10% β-1,6 glucans and 1.5–6% chitin [4], and the mannoproteins form a complex network with the β-glucans. Example of the mannoproteins include agglutinins (Agα1p, Aga1p and Aga2p), Flo1p, Sed1p, Cwp1p, Cwp2p, Tip1p and Tir1p/Srp1p. These proteins contain GPI residues that can be used as anchors for displaying target proteins on the cell surface. The mannoproteins are divided into two types based on their ability to be extracted by either sodium dodecyl sulfate (SDS) or β-glucanase. The former are extracted by non-covalent interactions while the latter are extracted by glucanase activity at β-1,3 and β-1,6 positions of the glucans. To engineer yeasts for cell surface display, the GPI-anchors are used to link a target heterologous proteins at either an N- or C-terminus [5, 6]. The N-terminal fusion method places a target protein at the N-terminus of a recombinant protein in a “target protein-anchor protein” pattern while the C-terminal fusion method places a target protein at the C-terminus in an “anchor protein-target protein” pattern [5]. In *S*. *cerevisiae*, a number of cell wall proteins such as Agα1p, Cwp1p, Cwp2p, Flo1p, Sed1p, Tip1p, Tir1, and YCR89w can be used as anchors for N-terminal fusion while Aga2p, Pir1p, and truncated Flo1p are used for C-terminal fusion [3].

The methylotrophic yeast *P*. *pastoris* (commonly used for heterologous protein expression) can utilize methanol as its sole carbon source. It is suitable for the production of recombinant proteins due to its high growth rate and the presence of a strong inducible *AOX1* promoter to control expression of a heterologous gene [7]. *P*. *pastoris* surface display has been successfully engineered using the GPI-anchor signal of Agα1p or Sed1p for N-terminal fusion and Aga2, Flo1p, or Pir1p for C-terminal fusion [5]. *P*. *pastoris* cells have been engineered to display recombinant proteins for use variously as whole-cell biocatalysts [8] in the food industry [9, 10], in wastewater treatment [11] and for oral administration to animals [12, 13].

Shrimp cultivation has been economically important in Thailand for many years. Beginning in 1980, production continually increased and the value of shrimp exports grew. In the 1990’s production was reduced by viral infections, first by yellow head virus (YHV) [14, 15] and then by WSSV [16]. The latter was initially (mistakenly) called a non-occluded baculovirus [14] and was later assigned to a new family *Nimaviridae* and genus *Whispovirus* [17]. WSSV has a rod to elliptical shape (80–120 x 250–380 nm). It is the most serious viral pathogen for all cultivated penaeid shrimp and it has caused economic losses in most countries where they are cultivated. The gross signs of infection include lethargy and the presence of white spots of 0.5 to 2.0 mm or more in diameter within the cuticle, first in the region of the cephalothorax an then all over the body. The virion is composed of more than 40 viral structural proteins including around 30 viral envelope proteins, 7 nucleocapsid proteins and 5 tegument proteins which have been identified by mass-spectrometry and immunogold electron microscopy [18, 19]. The viral genome is a ~300 kb double-stranded circular DNA molecule comprising 184 major open reading frames and 9 homologous regions [20].

The WSSV envelope contains four major proteins (VP19, VP24, VP26 and VP28) [21, 22] and two minor proteins VP37 [23] and VP76 [24]. A 3D model of the WSSV envelope protein was predicted from protein complexes formed and analyzed by coimmunoprecipitation and yeast two-hybrid assays [25]. The major protein 28 kDa (VP28) is the most abundant surface protein. It was first identified as an envelope protein by van Hulten *et al*., 2000 [26]. Transcriptional analysis revealed that VP28 had an A/T-rich promoter motif, a transcription initiation site 33 nucleotides upstream of the start codon and polyadenylation sites 15–16 nucleotides downstream of the stop codon [27].

Interaction of between viral binding proteins and virus particles plays an important role in WSSV infection as summarized by Sritunyalucksana *et al*. in 2012 [28]. For example, it has been shown by a pull down assay and by shrimp cell histology methods that VP28 forms a complex with *Pm*Rab7 and can protect them against WSSV [29, 30]. This binding occurs (as with Rab7 in some other viruses) via the endocytosis pathway [31–33]. Heterologously produced VP28 (rVP28) itself has been shown to protect *P*. *mododon* [34, 35], *Penaeus (Marsupenaeus) japonicus* [36], and the American crayfish *Procambarus clarkii* against WSSV [37–39] presumably via VP28 blocking of host cell receptors. So have VP28 long double-stranded RNAs (dsRNAs) for *Penaeus chinensis* [40] and *P*. *monodon* [41] by blocking VP28 formation in host cells. If yeast cells are used as the vehicle for such recombinant proteins or dsRNAs, it is possible that they may provide additional protection in the form of adjuvant activity arising from yeast mannans and β-glucan [42–47]. For example, yeast with a surface display of heterologously-produced 380R antigen has been applied as an oral vaccine against red sea bream iridovirus (RSIV) in the cultured marine fish *Pagrus major* [48], and surface displayed haemolysin virulent factor from *Vibrio harveyi* has been used as a vaccine in marine fish [49].

The aim of this study was to prepare separate yeast cell lines of the methylotrophic yeast *P*. *pastoris* for surface-based display of recombinant *Pm*Rab7 and VP28 so they might be tested in further studies for their efficacy in protecting shrimp against WSSV by oral delivery. To prepare the relevant clones, the GPI-anchoring domain was directly fused to each of the proteins so both could be displayed via yeast α-agglutinin (Agα1) following a protocol that has previously been successfully applied [3].

## Materials and Methods

### Strains, media and plasmids

*Escherichia coli* DH5α (*F* ^-^ Φ80d*lac*ZΔM15 Δ(*lac*ZYA-*arg*F) U169 *rec*A1 *end*A1 *hsd*R17 (r_k-,_ m_k+_) *pho*A *sup*E44 λ^-^ *thi*-1 *gyr*A96 *rel*A1) was cultivated in Luria Bertani medium (LB: 0.5% yeast extract, 1% tryptone, and 1% sodium chloride) at 37°C for 16 h. *E*. *coli* transformants were selected on LB agar supplemented with 100 μg/mL ampicillin. *S*. *cerevisiae* BY4742 (*MATα his3-Δ1 leu2-Δ0 lys2-Δ0 ura3-Δ0*) and *P*. *pastoris* KM71 (*aox1*::*ARG4*, *arg4*, *his4*) were routinely cultivated in yeast peptone dextrose medium (YPD: 1% yeast extract, 2% peptone, and 2% dextrose) at 30°C for 2–3 days. *P*. *pastoris* transformants were selected on minimal dextrose agar [MD: 1.34% yeast nitrogen base (YNB), 2% dextrose, 0.00004% biotin, and 2% agar], and on minimal methanol agar (MM: 1.34% YNB, 2% dextrose, 0.00004% biotin 1% methanol, and 2% agar) for verification of Mut^S^ transformants. For gene expression, they were grown in buffered minimal glycerol yeast extract broth [BMGY: 1% yeast extract, 2% peptone, 1.34% YNB, 0.00004% biotin, 1% glycerol and 100 mM potassium phosphate (pH 6.0)] and buffered minimal methanol yeast extract broth [BMMY: 1% yeast extract, 2% peptone, 1.34% YNB, 0.00004% biotin, 1% methanol and 100 mM potassium phosphate (pH 6.0)]. Plasmids pAO815 and pPIC9K were obtained from Invitrogen while plasmids pET17b-VP28 and pGEX4T1-*Pm*Rab7 were provided by Kallaya Sritunyalucksana [29].

### *In silico* analysis of *Pm*Rab7 and VP28 gene

In order to express *Pm*Rab7 and VP28 on the yeast cell surface using the anchoring domain of 3’-*AGα1* (Accession No. AAA34417.1), nucleotide and amino acid sequences of *Pm*Rab7 (Accession No DQ231062.1) and VP28 (Accession No. AF440570) genes were analyzed using vector NTI11. 5 program and SMART database, respectively. For these nucleotide sequences, the restriction sites *Bgl*II, *Eco*RI, *Sma*I and *Not*I could not be allowed, and if they did occur, they had to be removed using polymerase chain reaction (PCR) methods. With respect to the amino acid sequences, any secretion signal peptides or transmembrane domains of *Pm*Rab7 and VP28 had to be compared to the GPI-anchoring domain of α-agglutinin so that any regions of homology (if found) could be removed.

### Construction of *P*. *pastoris* cell surface display plasmids

A *Sac*I—*Xba*I DNA fragment (1,560 bp) containing α-factor secretion signal from pPIC9K was cloned into a pAO815 plasmid at the corresponding site to generate a pAOα plasmid. The region comprising nucleotides 991–1,953 and containing the GPI-anchoring domain of *AG*α1 was amplified by high fidelity *Pfu* DNA polymerase using primers *AGα*1-*Sma*I F and *AGα*1-*Not*I R with *S*. *cerevisiae* BY4742 genomic DNA as the template. A DNA fragment comprised of nucleotides 64–666 of *Pm*Rab7 was amplified from plasmid pGEX4T1-*Pm*Rab7 by a 2-step PCR method. First, the N-terminus of *Pm*Rab7 was amplified without a start codon using primers m*Pm*Rab7-*Eco*RI F and m*Pm*Rab7-*Bgl*II_1 R. Second, the C-terminus of *Pm*Rab7 was amplified without a Cysteine-containing motif (CSC motif) and without a *Bgl*II site, using primers m*Pm*Rab7-*Bgl*II_2 F and m*Pm*Rab7-*Sma*I R. Finally, the two fragments were fused to generate the full length m*Pm*Rab7 using primers m*Pm*Rab7-*Eco*RI F and m*Pm*Rab7-*Sma*I R. A fragment of the WSSV-VP28 gene comprising nucleotides 82–612 (without a signal peptide, a transmembrane domain at the 5’-end and a stop codon) was generated using primers VP28-*Eco*RI F and VP28-*Sma*I R. Finally, the expression plasmids with gene fusions of α factor-m*Pm*Rab7-*AG*α1 (pAOα-RA), α factor-pVP28-*AG*α1 (pAOα-VA) and α factor-*AG*α1 (pAOα-A) for expression control were constructed by insertion of amplified fragments of m*Pm*Rab7 or pVP28 and *AG*α1 into pAOα. All the specific primers used for construction of the cell surface display plasmids are listed in Table 1.

**Table 1:** Lists of DNA primers used in this study.

**Name**	**Sequences** ^a^	**Purposes**
m*Pm*Rab7-*Eco*RI F	CTCGAATTCGCATCTCGCAAGAAGATTCTC	To amplify N-terminal region of *Pm*Rab7
m*Pm*Rab7-*Bgl*II_1 R	CTGCATTGTGACCAATCTGTCATCAACC	
m*Pm*Rab7-*Bgl*II_2 F	GGTCACAATGCAGgTCTGGGATACAGCTGG	To amplify C-terminal region of *Pm*Rab7 and modified *Bgl*II site
m*Pm*Rab7-*Sma*I R	CTCCCCGGGTGCATCCTGTTTAGCCTTGTT	
VP28-*Eco*RI F	CTCGAATTCAGGTATCACAACACTGTGACCAAG	To amplify partial VP28 without signal peptide and transmembrane region
VP28-*Sma*I R	CTCCCCGGGCTCGGTCTCAGTGCCAGAGTA	
*AGα1*-*Sma*I F	CTCCCCGGGAGCGCCAAAAGCTCTTTTATC	To amplify *3’AGα1* gene with anchoring domain
*AGα1*-*Not*I R	CTCGCGGCCGCTTAGAATAGCAGGTACGACAA	
5’*AOX1* F	GACTGGTTCCAATTGACAAGC	To confirm KM71 transformants at the *AOX1* locus
3’*AOX1* R	GCAAATGGCATTCTGACATCC	

^a^Underlined letters indicate restriction recognition sequences (GAATTC = *Eco*RI; CCCGGG = *Sma*I; GCGGCCGC = *Not*I; AGgTCT = modified *Bgl*II site with altered nucleotide shown in lower case.

### Yeast transformation

Transformation of *P*. *pastoris* KM71 was achieved by electroporation according to the *Pichia* expression kit manual (Invitrogen) [50]. His^+^ transformants were selected on MD plates and Mut^S^ phenotype was verified on MM plates. Correct integration of the *AOX1* cassette with α factor-m*Pm*Rab7*–AGα1*, α factor-pVP28-*AGα1* and α factor-*AGα1* was confirmed by colony PCR using *AOX1* primers (Table 1).

### Expression of the m*Pm*Rab7-*AG*α1 and pVP28-*AG*α1 genes

Recombinant yeasts were pre-cultured in BMGY at 30°C with shaking at 250 rpm for 16–18 h. Then the pre-cultures were transferred into fresh BMGY under the same conditions for high cell growth. Cells were harvested and washed twice with PBS (pH 7.4) at room temperature. The cell pellet was then re-suspended in one-fifth volume of BMMY containing 1% methanol and 2% casamino acids for further growth at 20°C at 250 rpm for several days. In order to maintain induction, 100% methanol was added to 1% final concentration every 24 h.

### Immunofluorescent labeling of recombinant yeast

Immunofluorescence labeling of recombinant yeasts was used for examination of yeast cell expression of m*Pm*Rab7 or pVP28 on the cell surface according to a method modified from Wasilenko *et al*. (2010) [13]. Induced cells were washed twice with PBS containing 1 mM PMSF. Cells at a concentration of 1×10^7^ were incubated in PBS containing 4% BSA with diluted polyclonal anti-rabbit *Pm*Rab7 or VP28 antibody (1:500) (Biomedical Technology Research Unit, Faculty of Associated Medical Sciences, Chiang Mai University) at room temperature for 1 h. Cells were then washed with the same buffer three times and incubated with goat anti-rabbit IgG conjugated-Alexa Fluor 488 antibody (1:1,500) (Invitrogen). After washing with the same protocol, 30 μL of the fluorescence-labelled cells were dropped on a glass slide, air dried and fixed with 100% acetone for 3 min. The fixed cells were then examined under a confocal laser scanning fluorescence microscope (CLSM; Olympus Fluoview FV1000). A recombinant yeast transformed with an empty vector was used as a negative control. Photographs were taken using a magnification of 1,800×.

### Quantification of surface-expressed proteins by ELISA

Yeast cells expressing m*Pm*Rab7-Agα1p or pVP28-Agα1p were assayed using a modified, previously published ELISA protocol (2001) [51]. Briefly, 10^8^ yeast cells (OD_600_ = 10) were re-suspended in 100 μL of a serially diluted primary polyclonal rabbit antibody against *Pm*Rab7 or VP28 (0, 1, 10, 100, 250, 500, 750, and 1,000 μg/ml) in PBS containing 1% BSA and incubated at room temperature for 1 h. Then, cells were washed 3 times in PBS. Goat anti-rabbit IgG antibody conjugated with horseradish peroxidase (1 mg/ml) (Invitrogen) was added and the mixture was incubated at room temperature for 1 h. After washing in the same way, the cells were re-suspended in 100 μL of PBS and subjected to protein surface detection by incubating in 100 μl of HRP substrate 3,3’,5,5’-tetramethylbenzidine (TMB) (Sigma) in the dark for 30 min followed by addition of 100 μl of 2 N H_2_SO_4_ to stop the reaction. The yeast cell suspension was spun down and the supernatant OD_450_ was measured using a microplate reader.

### Competitive ELISA to verify specific binding by surface expressed proteins

Sandwich ELISAs were performed by using yeast cells (OD_600_ = 10) displaying m*Pm*Rab7 or pVP28 and bound to purified, expressed *Pm*Rab7 and VP28, respectively. The purified proteins prepared from *E*. *coli* harboring pET17b-VP28 [29] and pET17b-*Pm*Rab7 [29] (10, 10^1^, 10^2^, 10^3^, 5×10^3^, 10^4^, and 2×10^4^ ng/ml) were added to PBS containing 1% BSA and incubated at room temperature for 1 h. Then, cells were washed 3 times in PBS and further incubated, as appropriate, with polyclonal rabbit *Pm*Rab7 or VP28 primary antibody (as appropriate) at room temperature for 1 h. Subsequent steps were the same as those described for the ELISA assay above.

### Yeast agglutination and inhibition assays

Since our two surface-expression yeast clones produced complementary binding proteins, it was predicted that they would agglutinate as a result of this binding. To test this prediction, the complementary yeast cells were mixed in PBS containing 1% BSA to obtain an initial OD_600_ = 0.7 and then they were left to stand for 1 h. Agglutination was measured by the change in OD after 1 h. For the control, yeast cells expressing m*Pm*Rab7 or pVP28 were mixed with yeast cells expressing Agα1p. The experiment was performed in three independent replicates and percentage of agglutination was calculated by the formula:

$$\%\text{Agglutination}=\frac{\text{OD}600_{0\text{min}}-\text{OD}600_{60\text{min}}}{\text{OD}600_{0\text{min}}}\times 100$$

Tests to measure the blocking of agglutination by polyclonal rabbit anti-*Pm*Rab7 or anti-VP28 were also carried out. After incubation with their respective antibodies in PBS containing 1% BSA for 1 h, the cells were washed 3 times with the same buffer and then mixed as above. Agglutination was compared as above, with that of cells without pre-incubation. The experiment was performed in three independent replicates and percentage of agglutination inhibition was calculated by considering agglutination of the untreated cells as 100%.

### Flow cytometry

The fluorescence labeled cells suspended in PBS containing 1 mg/ml BSA were measured using a FACScanto instrument (BD Biosciences). Briefly, yeast cells were incubated in the appropriate diluted antibody (1:500) with gentle shaking for 1 h at room temperature. After washing with PBS containing 1 mg/ml BSA three times, cells were incubated with goat anti-rabbit IgG conjugated-Alexa Fluor 488 antibody (1:1,500). Yeast cells expressing m*Pm*Rab7-Agα1p or pVP28-Agα1p were detected by Fluorescence channel 1 (FL1 channel) using 530/30-nm band pass filter. The labeled cells were counted and events of cells vs. log of fluorescence (FL1 log) were plotted. Fluorescence of non-labeled cells and negative controls severed gave background values. Data analysis of fluorescence intensity of cells was correlated by number of cells vs. FL1 log. The experiment was performed in three independent replicates.

## Results and Discussion

### Construction of expression plasmids harboring m*Pm*Rab7- *AGα1* or pVP28-*AGα1*

Analysis of the nucleotide and amino acid sequences of *Pm*Rab7 for suitability in yeast cell surface expression revealed that *Pm*Rab7 had one inappropriate *Bgl*II site and an inappropriate prenylation motif “CSC”. Removal of the inappropriate *Bgl*II site at position ^179^AGATCT^184^, was achieved by modifiying A^181^ to G^181^ by two-step PCR. This also changed the amino acid at that position from isoleucine (I) to valine (V), both of which are hydrophobic. The “CSC” motif (involved in membrane-association localization and function) [52, 53] and similar to the GPI-anchor domain of α-agglutinin in yeast, had to be eliminated in order to fuse *Pm*Rab7 with yeast GPI. In addition, the start and stop codons of *Pm*Rab7 were eliminated because the N-terminus had to be fused to the yeast α-factor secretion signal and then to the cloning vector, while the C-terminus had to be fused to *S*. *cerevisiae* 3’-*AGα1*. The modified *Pm*Rab7 for cell surface display (without a *Bgl*II site, prenylation motif or start/stop codons) was named m*Pm*Rab7.

VP28 contained a signal peptide and a transmembrane domain at its N-terminus as described in a previous report [25]. This 21 amino acid signal peptide (^1^MDLSFTLSVVSAILAITAVIA^21^) is involved in transportation of the VP28 protein into the viral particle membrane while the 23 amino acid transmembrane domain (^5^FTLSVVSAILAITAVIAVFIVIF^27^) has been implicated in maintenance of viral structure. These domains overlapped at amino acids 5–21. In order to clone VP28 without these 2 regions, only nucleotides 82–612 were amplified and called pVP28.

**Fig 1:**
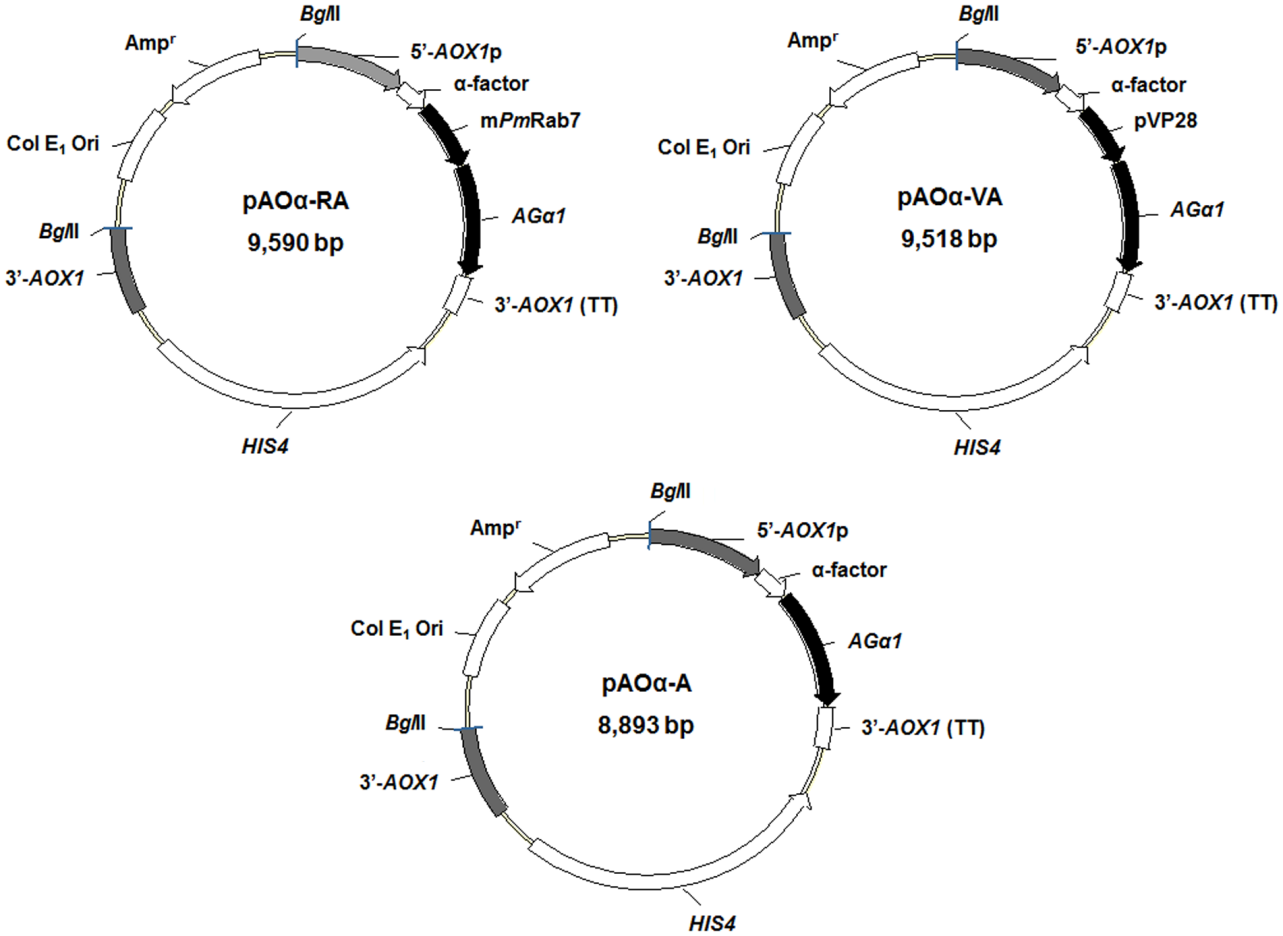
Physical maps of plasmids harboring m*Pm*Rab7 and pVP28 fused with half 3’-agglutinin of *S*. *cerevisiae* for surface display by *P*. *pastoris*.

In order to express m*Pm*Rab7-*AGα1* and pVP28-*AGα1* on the cell surface, the proteins needed appropriate secretion signals. Thus, we first created the vector pAO815 with a α-factor secretion signal and called it pAOα. This was used to insert m*Pm*Rab7-*AGα1* or pVP28-*AGα1* and with *AGα1* alone as a control to obtain plasmids pAOα-RA, pAOα-VA and pAOα-A, respectively (Fig 1).

Previous reports on *P*. *pastoris* surface display have used the drug resistance gene Kan^r^ as a selectable marker in yeast and have sometimes also included Amp^r^, depending on how the recombinant plasmid was linearized prior to transformation. Most of these constructs were developed for bioremediation applications or as whole-cell biocatalysts [8]. For applications such as vaccines, an example is surface display of highly pathogenic avian influenza virus hemagglutinin as an oral vaccine in chickens [13]. However, the developed yeast contained Kan^r^ and Amp^r^ genes. Cell surface display of phytase for application as a feed supplement was also reported, and the recombinant yeast used harbored the resistance antibiotic gene Zeo^r^ as a marker [12]. Potential applications of cell surface displays for oral vaccines in animals or as biodrugs for human have mainly employed *S*. *cerevisiae* hosts that do not contain drug resistance selectable markers. Our development of a target gene expression-cassette model for cell surface display in *P*. *pastoris* without drug resistance selectable markers is the first report of a technique that could be applied for oral administration of bioactive proteins in shrimp.

### Confirmation of m*Pm*Rab7 and pVP28 surface expression by confocal microscopy

**Fig 2:**
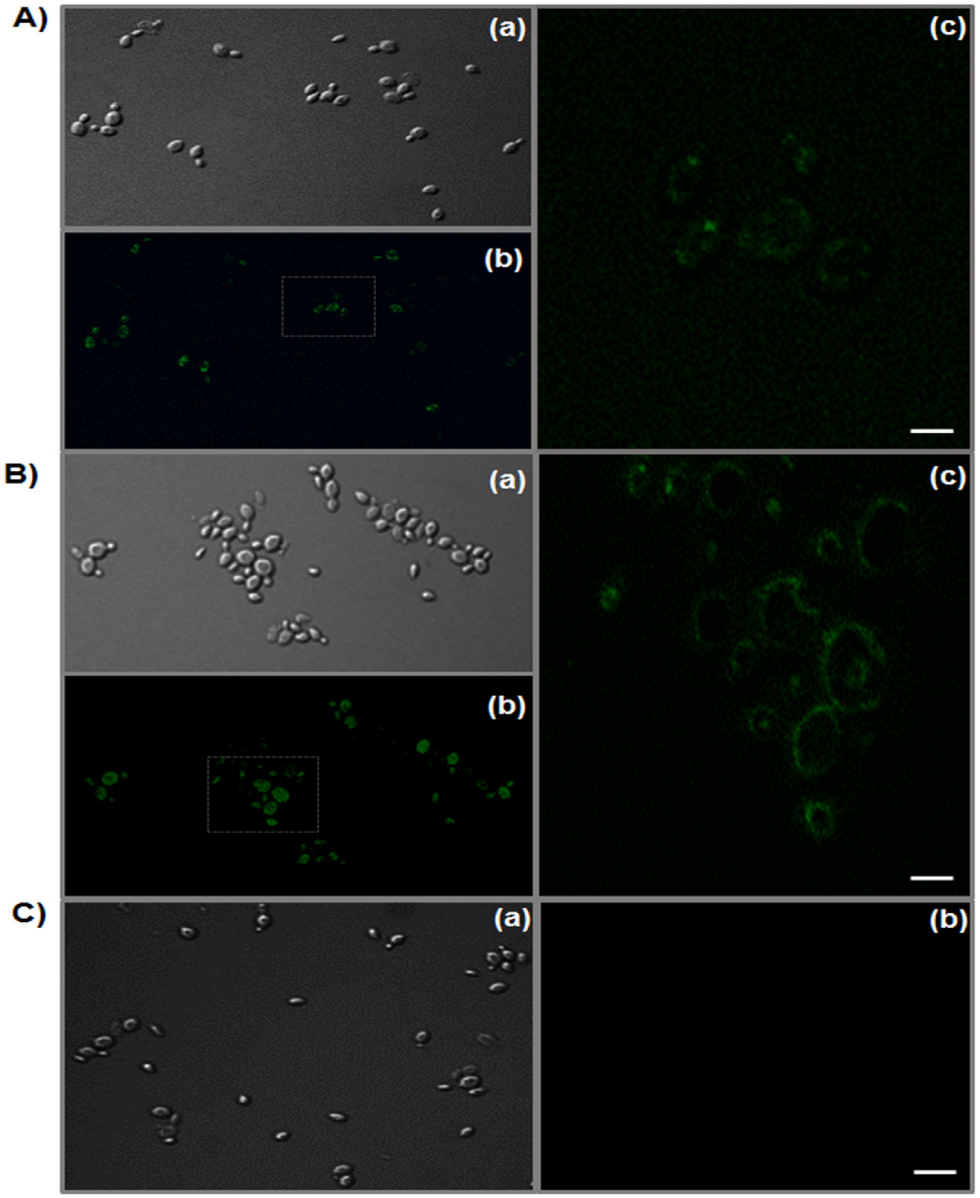
Immunofluorescence microscopy of m*Pm*Rab7-Agα1p and pVP28-Agα1p surface displayed by *P*. *pastoris*. Methanol induced His^+^Mut^S^ transformants with (A) m*Pm*Rab7-Agα1p, and (B) pVP28-Agα1p grown at 20°C for 96 h and incubated with polyclonal rabbit anti-*Pm*Rab7 and anti-VP28, respectively. Panel (a) is light microscope, (b) and (c) are fluorescent microscope where (c) is the enlargement of some group of cells in (b). Fluorescence was observed on the cell surface more than in (C) the Agα1p control. Bar = 5 μm, fluorescence magnification = 1,800 ×.

The production of recombinant protein in a foreign organism can lead to improper folding and localization. To confirm proper folding, secretion, and targeting of the expressed m*Pm*Rab7-Agα1p and pVP28-Agα1p fusion proteins to the cell surface, confocal laser scanning fluorescent microscopy (CLSM) was carried out to visualize the location of the fusion proteins. Staining of the m*Pm*Rab7-Agα1p and pVP28-Agα1p expressing cells (induced at 20°C for 96 days) with respective antibodies revealed their presence on the cell surface and indicated that the respective fusion proteins were correctly attached to the yeast cell walls (Fig 2). The control cells expressing *AGα1*gave no significant fluorescence in these tests. These results confirmed the specificity of the immunofluorescence labeling system and correct localization of the fusion protein on the cell wall. Fluorescence microscopy has been widely used to examine *P*. *pastoris* cell surface proteins [13, 54–56].

### Quantification of the surface-expressed proteins using specific antibodies

**Fig 3:**
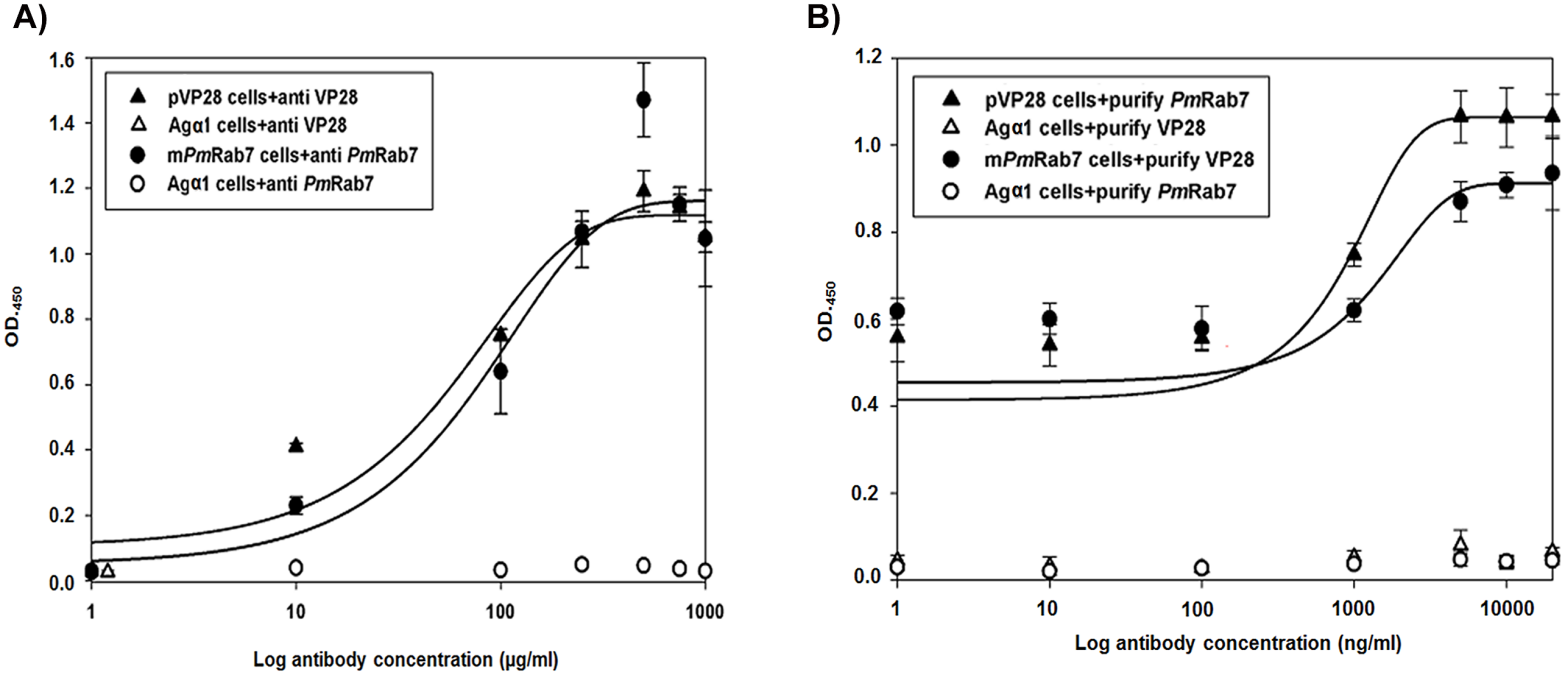
Quantification of m*Pm*Rab7 and pVP28 on the cell surface of *P*. *pastoris* by (A) indirect ELISA and (B) sandwich ELISA. The quantity of methanol-induced His^+^Mut^S^ yeast cells was 10^8^ cells/ml (OD_600_ = 10). The values were obtained from three independent experiments. Bar = means ± SD.

When increasing concentrations of polyclonal anti-*Pm*Rab7 and anti-VP28 antibodies were used against10^8^ yeast cells/ml expressing m*Pm*Rab7 and pVP28, respectively, it was found that they displayed m*Pm*Rab7 and pVP28 at approximately 250 μg on the cell surface under the methanol induced conditions used (Fig 3A). When the concentration of antibodies was increased beyond this point, the optical densities were more or less stable suggesting that expression of these proteins on the cell surface was at its saturation limit at 250 μg/mL. This yeast-ELISA method was a high-throughput one to detect surface proteins without the need for protein extraction and purification [57]. The same technique was employed for quantification of ZZ domain expression of *Staphylococus aureus* protein on the cell surface of *S*. *cerevisiae* [51].

### Specific binding of surface expressed proteins confirmed by competitive ELISA

When 10^8^ yeast cells/ml expressing m*Pm*Rab7 at 250 μg/ml were pre-incubated with various concentrations of VP28 protein (1 to 7,500 ng/mL) before exposure to anti-VP28 polyclonal antibody, saturation binding was observed between 7,500 to 20,000 ng/ml (Fig 3B). In the reverse reaction with 10^8^ yeast cells/ml expressing pVP28 at 250 μg/ml and pre-exposed to *Pm*Rab7, saturation binding was observed at the same concentration (Fig 3B). By contrast, Agα1-expressing control cells did not bind either *Pm*Rab7 or VP28. The results revealed specificity of the interaction between free, unmodified *Pm*Rab7 or VP28 and their respective cell-bound but modified binding partners. This result corresponded to the binding of free human serum albumin (HSA) to ZZ domain of *S*. *aureus* on the cell surface of *S*. *cerevisiae* [51]. Our results implied that that the structure of expressed m*Pm*Rab7 and pVP28 on the cell surface of *P*. *pastoris* folded correctly and that they retained their ability to bind with partner proteins.

### Agglutination of yeast cells with complementary surface-expressed proteins

**Fig 4:**
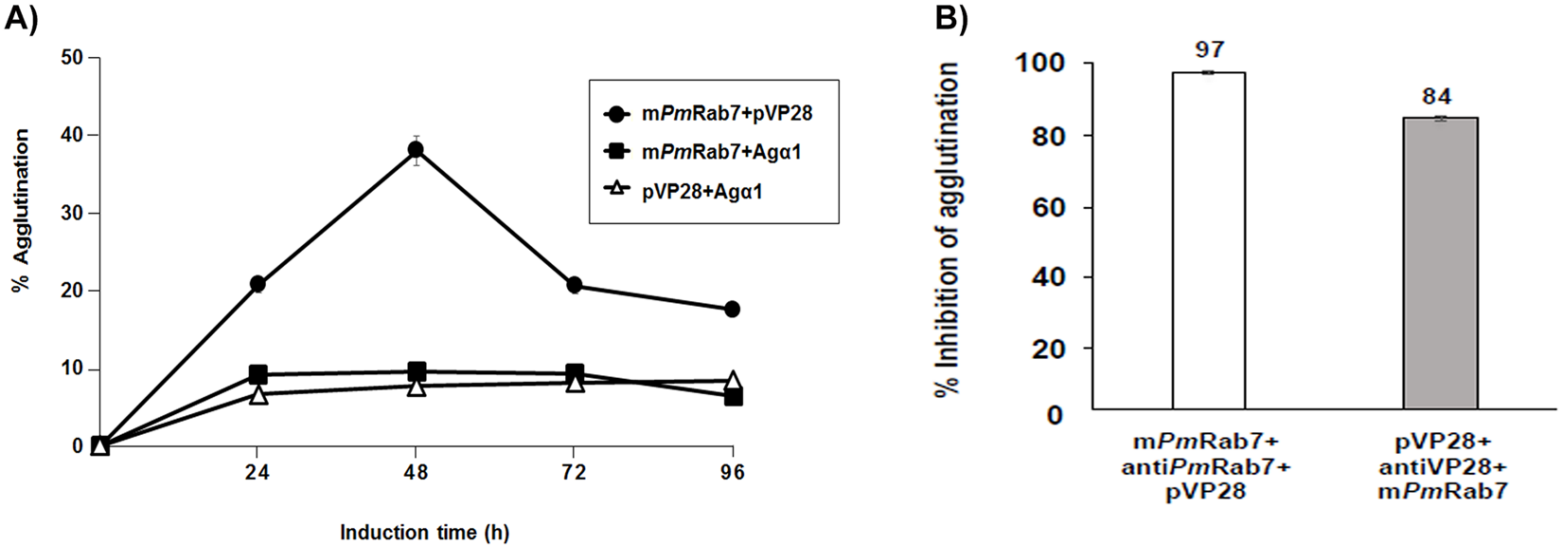
Yeast agglutination and agglutination inhibition assays. (A) OD_600_ of His^+^Mut^S^ yeast cells induced by methanol for 24–96 h, mixed at initial OD_600_ = 0.7 and measured after 1 h. Percent agglutination was calculated from OD_600_ at time zero and after 1 h. (B) Percent inhibition of agglutination by treatment of yeast cells with *Pm*Rab7 and VP28 antibodies for 1 h prior to agglutination assay. Percent inhibition was calculated as percent of agglutination with and without specific antibody treatment. The values were obtained from three independent experiments. Bar = means ± SD (Some of the SD bars are so small that they cannot be seen in the graph).

Yeasts can exhibit 4 types of cellular aggregation: sexual, flocculation, biofilm formation, and filamentous growth. Yeast aggregations usually occur via small chemical modifications of the cell wall in response to environmental changes. Sexual agglutination is mediated by a mannoprotein agglutinin by which a-cells interact with α-cells via a- and α –agglutinin on their cell surfaces [58]. Based on this principle, agglutination tests were carried out to further confirm the binding between m*Pm*Rab7 and pVP28. Since expression of heterologous proteins by yeast is time dependent [50], we need to determine the optimum induction time to obtain maximum surface expression of active m*Pm*Rab7 and pVP28. This would be an important issue in the production protocol for production of yeast cells intended for laboratory tests on their efficacy in protecting shrimp against WSSV infection. The results revealed maximum agglutination of 38% at 48 h induction time (Fig 4A) compared to only 20% agglutination with longer induction times when compared to no agglutination in mixtures of these cells with those expressing Agα1p. Thus, we selected cells induced for 2 days to study agglutination inhibition by addition of anti-*Pm*Rab7 or anti-VP28 to the complementary yeast cell mixtures. Results showed that addition of either anti-*Pm*Rab7 or anti-VP28 gave more than 90% inhibition (Fig 4B). These results confirmed those from the ELISA tests indicating that the surface expressed proteins retained their binding capacity.

### Quantitative expression of protein fusion analyzed by flow cytometry

**Fig 5:**
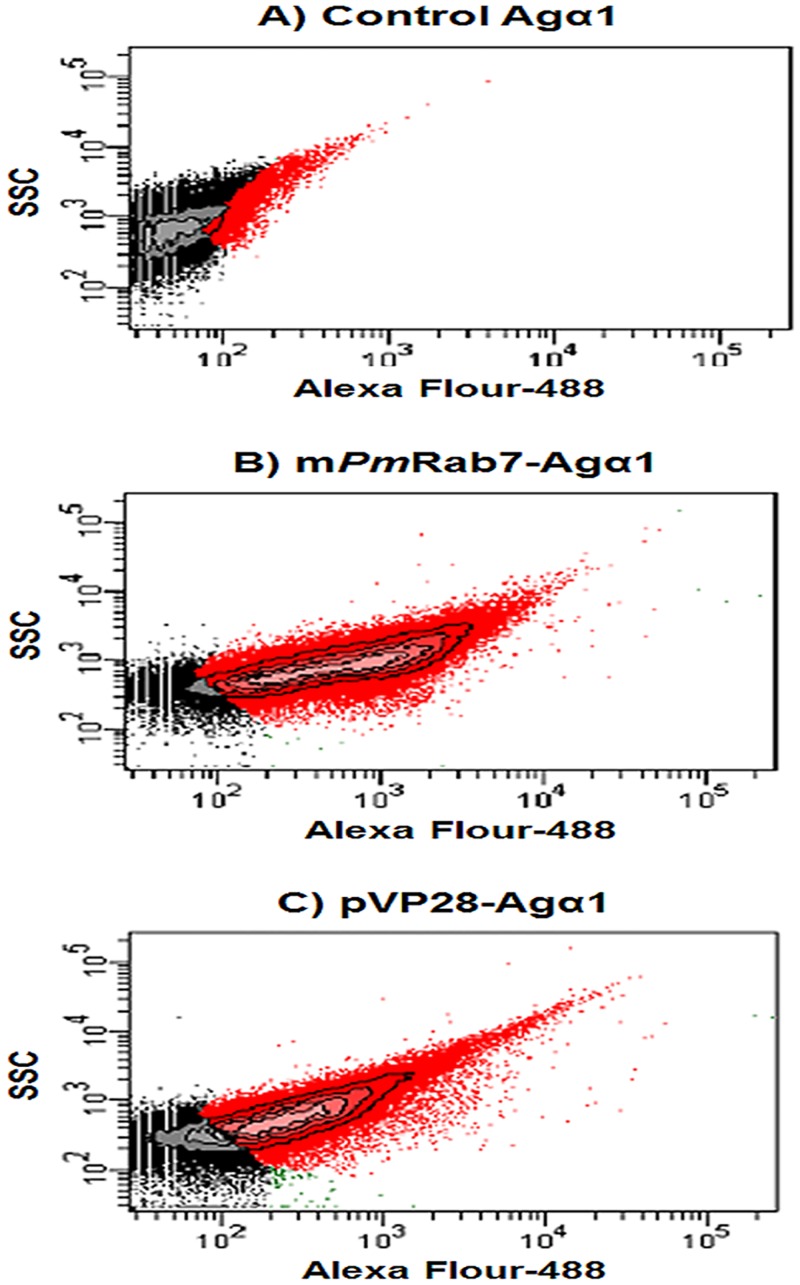
Cell flow cytometry results. (A) Results for negative control Agα1p only and showing approximately 20% autofluorescence. (B) Yeast cells expressing m*Pm*Rab7-Agα1p and showing fluorescence for approximately 90% of total events. (C) Yeast cells expressing pVP28-Agα1p and showing fluorescence for approximately 70% of total events. The data was selected by relative statistical value from three independent experiments.

In order to quantify the displayed recombinant proteins, fluorescence on the cell surface of transformants was measured by flow cytometry using anti-*Pm*Rab7 and anti-VP28 antibody. In three independent experiments each with a total of 10^5^ events, it was revealed that approximately 90% and 70%, respectively, of the total events involved cells expressing m*Pm*Rab7 and pVP28 on the yeast surface. Non-specific fluorescence detected in the control strain was about 20% of total events (Fig 5). The fluorescence intensity for m*Pm*Rab7-Agα1p and pVP28-Agα1p was significantly higher in the test cells than in the non-stained cells and in the stained Agα1p cells. In our case, we constructed the surface display by fusion of the target genes with the GPI-anchoring domain of Agα1. To improve the proportion of m*Pm*Rab7 and pVP28 positive surface display cells, other GPI-anchoring domains may be explored. In addition, cultivation time may also be optimized to improve protein display.

## Conclusions

In this study two methylotrophic *P*. *pastoris* yeast cell clones were engineered, one to express the shrimp viral receptor protein *Pm*Rab7 and the other its viral binding partner WSSV-VP28 on the cell surface. Both proteins had previously been demonstrated to protect shrimp against WSSV. Both were successfully displayed on its cell surface and shown to retain their mutual binding activities. These engineered cells are now ready to be tested for use as orally delivered antiviral agents to protect shrimp against WSSV infection.
